# Dual Modulatory Effects of Phytochemicals from *Iris ×germanica* L. var. *florentina* Dykes Rhizome Extract on Melanogenesis

**DOI:** 10.3390/molecules30173626

**Published:** 2025-09-05

**Authors:** Sunghan Yim, Lisa Rozga, Steve Missler, Dmitri Sitnikov, Xiaozhong Liu, Sudhir Baswan

**Affiliations:** Amway Research and Development, Ada, MI 49355, USA; lisa.rozga@amway.com (L.R.); smissler202@comcast.net (S.M.); dmitri.sitnikov@amway.com (D.S.); xiaozhong.liu@amway.com (X.L.); sudhir.baswan@amway.com (S.B.)

**Keywords:** *Iris ×germanica* L. var. *florentina* Dykes, melanogenesis, skin pigmentation, bioassay-directed (guided) fraction, LC-MS, extraction

## Abstract

Abnormal skin pigmentation can cause significant esthetic concerns and impact quality of life. As melanin determines pigmentation, melanogenesis is a key target to manage pigmentation disorders. This study investigated the effects of extracts from the rhizome of *Iris ×germanica* L. var. *florentina* Dykes (often called *Iris florentina* L.) on melanogenesis. Active phytochemicals were identified by combining LC-MS-MS metabolic profiling with subsequent bioassay-directed fractionation of chromatographic eluent collected into 96-well plates. Fractions 41–43 increased melanin and contained germanaism B, providing evidence that it is a melanogenesis stimulator. In contrast, fractions 90–93 reduced melanin and contained iriflorental and iripallidal, identified as prospective melanogenesis inhibitors. To explore extract-based applications, the plant was subjected to ethanolic, chloroform, and supercritical carbon dioxide (SC-CO_2_) extractions and tested in skin equivalent tissues. The ethanolic extract (rich in germanaism B) increased pigmentation, while the chloroform extract (higher in iriflorental and iripallidal) decreased it. The SC-CO_2_ extract, with minimal germanaism B and enriched iriflorental and iripallidal, showed stronger depigmenting effect. This study is the first to report that rhizome of *I. florentina* contains phytochemicals with opposing effects on melanogenesis. Through different extraction processes, targeted extracts from a single botanical can address both hyper- and hypopigmentation, offering a novel approach to pigmentation modulation.

## 1. Introduction

Skin pigmentation varies widely in human populations due to the presence of a chemically inert pigment called melanin. The melanin is produced deep inside the skin and deposited in a mosaic manner at the surface of the skin [1,2], in which both the amount and distribution of melanin are responsible for skin color variation [3]. Besides its role in defining ethnicity and geographical origins of people, melanin has an essential function in protecting skin against ultraviolet (UV) radiation [4]. Although the melanin is critical for providing a self-defense mechanism against harmful environmental factors, there have been many studies on regulating the synthesis and transfer of melanin to ameliorate esthetic problems caused by hyper- or hypo-pigmentation of the skin [5,6,7]. The regulation of melanin synthesis is intricate, yet it has become an important strategy across skincare, dermatology, and pharmaceutical industries, driving the development of products that help reduce unwanted skin pigmentation [8]. Growing commercial interest in skin pigmentation modulation has also led to a diverse market for both skin darkening and lightening products.

Skin darkening is often desirable to improve appearance or create a more even skin tone. Tanning, commonly achieved through UV exposure, increases skin darkening but also accelerates skin aging and raises the risk of skin cancer [9,10]. Therefore, the ability to generate darker skin tone without incurring photo-damage has led to development of “self-tanning” applications [11]. Dihydroxyacetone (DHA)-based products and bronzers are widely used to give the appearance of a tan without affecting actual pigmentation [12]. Other strategies involve stimulating melanin production through melanogenesis pathways using synthetic or natural agents. For example, afamelanotide activates melanocyte-stimulating hormone (α-MSH)/melanocortin 1 receptor (MC1R) pathway [13], while Salt-inducible kinase (SIK) inhibitors boost microphthalmia-associated transcription factor (MITF) and transient receptor potential cation channel subfamily M member 1 (TRPM1) independently [14]. Compounds like forskolin and 3-Isobutyl-1-methylxanthine (IBMX) enhances melanin synthesis by modulating cyclic adenosine monophosphate (cAMP) signaling downstream of α-MSH/MC1R [15,16,17]. Many darkening agents were also isolated from numerous plant sources such as *Glycyrrhiza glabra*, *Vigna angularis*, citrus plants, etc., which induce melanogenesis by stimulating melanogenic protein expression and pathways in both in vitro or in vivo systems [2,18].

On the other end of the spectrum, skin lightening products are commercially available for cosmetic purposes to achieve a lighter skin tone and are widely used to address hyper-pigmentary conditions such as melasma, dark spots, and solar lentigo [19,20]. As skin pigmentation is mainly determined by the content of melanin in melanosomes and the distribution of melanosomes in keratinocytes, the down-regulation of melanogenesis and melanosome transfer to keratinocytes are often contemplated as primary targets for skin lightening agents [21]. Tyrosinase, a rate-limiting enzyme in melanin synthesis, is a key target for many inhibitors, including hydroquinone [22], L-ascorbic acid [23], kojic acid [24], arbutin [25], azelaic acid [26], ellagic acid [27], tranexamic acid [28], resorcinol derivatives [29]. In addition, melanosome transfer inhibitors such as protease-activated receptor 2 (PAR-2) inhibitors [30], niacinamide [31], and cytidine [6,32] have demonstrated skin lightening effects without impacting melanin synthesis.

*Iris ×germanica* L. var. *florentina* Dykes, often called *Iris florentina* L. [33], belonging to the family Iridaceae, is a white-flowered variant of *Iris germanica* [34] and is distributed predominantly along the Mediterranean coast of Europe, including France, Spain, and Italy [35]. Its rhizome, commonly known as the orris root have been used as a perfume ingredient and fixative in aromatherapy and traditionally in Iranian medicine as an insecticide and repellent [36]. Phytochemical investigations of *I. florentina* have revealed a variety of secondary metabolites. Prior studies have isolated norisoprenoids, xanthone glucosides, and flavone glucosides from the plant [37,38]. More recent research has focused on isoflavonoid constituents, leading to the identification of novel isoflavone glucosides and evaluations of their bioactivity, including cytotoxicity and inhibitory effects on advanced glycation end products [38,39]. Despite these studies, systematic investigations of *I. florentina* in dermatology, particularly regarding its effects on skin pigmentation, remain limited.

In some cultures, a tanned skin tone is considered desirable, while in others, a lighter skin tone is traditionally associated with beauty [40]. Along with an increased demand for plant-based ingredients in the last few decades, there is an opposing trend that rejects synthetic counterparts in the skin care industry. In this context, we report for the first time that extracts of *I. florentina* rhizome demonstrate dual effects on melanogenesis, exhibiting both stimulatory and inhibitory activity. Specifically, germanaism B was identified as a melanin synthesis-inducing compound, whereas iriflorental and iripallidal were found to inhibit melanogenesis. These findings highlight *I. florentina* rhizome as a versatile botanical resource for developing both skin-lightening and skin-darkening agents.

## 2. Results and Discussion

### 2.1. Phytochemical Analysis

For screening purposes, metabolites were isolated from dried *I. florentina* rhizome by two different extraction methods: (A) using 70:30 ethanol/water and (B) using chloroform. Each method was selected for the ability to extract hydrophilic or lipophilic constituents, respectively. Both extracts were analyzed by Liquid chromatography–mass spectrometry (LC-MS) to characterize their phytochemical composition. Analysis revealed the presence of many compounds, and the ten most abundant peaks common to both extracts were characterized. These consisted primarily of flavonoids, including various isoflavones and their glycosides. Identification was based on chromatographic retention time, UV-Vis spectrum, and mass spectrum of each peak. Accurate mass analysis was also performed to verify elemental composition within 5 ppm mass error.

**Figure 1:**
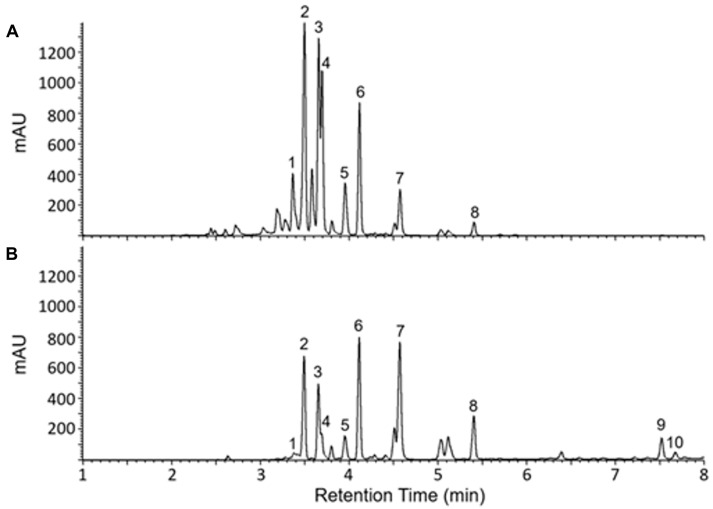
UV-Vis total wavelength chromatograms of *I. florentina* rhizome 70:30 ethanol/water extract (**A**) and chloroform extract (**B**) metabolite profile comparison. Numbered peaks are listed in Table 1.

The identified compounds include Irisolone 4′-O-diglucoside, Iridin, Germanaism A, Germanaism B, Irilone 4′-O-glucoside, Irisolidone 7-O-glucoside, Irigenin, Irisolidone, Iriflorental, and Iripallidal (Figure 1 and Table 1). Some of these compounds have been reported to exhibit biological activities such as anti-inflammatory, antioxidant, α-amylase inhibitory, antiproliferative, cytotoxic, piscicidal, and antimicrobial effects [41,42,43,44,45,46,47]. However, the biological activities of several of these compounds remain largely uncharacterized [48] and require further investigation. Figure 1 shows the chromatograms of the 70:30 ethanol/water extract (A) and the chloroform extract (B). As illustrated, the 70:30 ethanol/water extract predominantly yielded more abundant hydrophilic compounds and did not show peaks corresponding to Iriflorental (9) and Iripallidal (10), which are more lipophilic. In contrast, these lipophilic compounds were clearly observed in the chloroform extract, which contained fewer, less abundant hydrophilic constituents. The identified compounds are listed in Table 1.

### 2.2. Identification of Melanogenesis-Modulating Phytochemicals via Bioassay-Direct Fractionation

**Figure 2:**
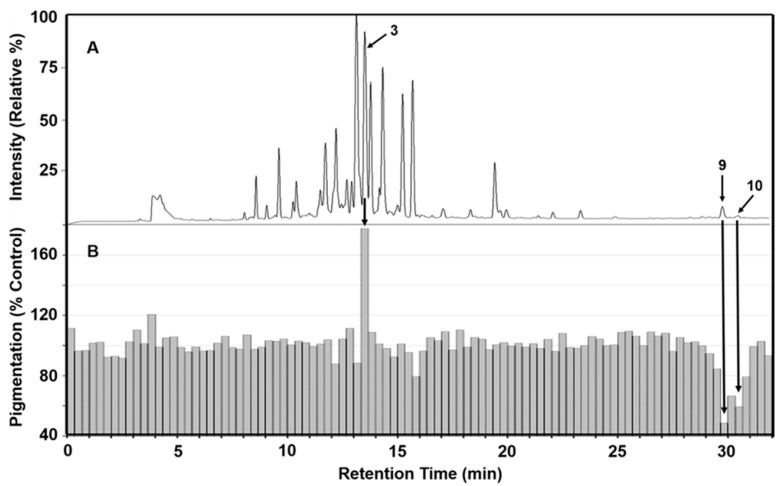
BDF results for a 90:10 ethanol/water extract of *I. florentina* rhizome. (**A**) LC-UV chromatogram at 260 nm showing phytochemical peak profile. (**B**) time-aligned bar graph (each bar representing a fraction from the LC effluent collected for 20 s), showing pigmentation responses using B16 cell culture assay. Arrows indicate identified compounds: (**3**) Germanaism B, (**9**) Iriflorental, and (**10**) Iripallidal.

**Figure 3:**
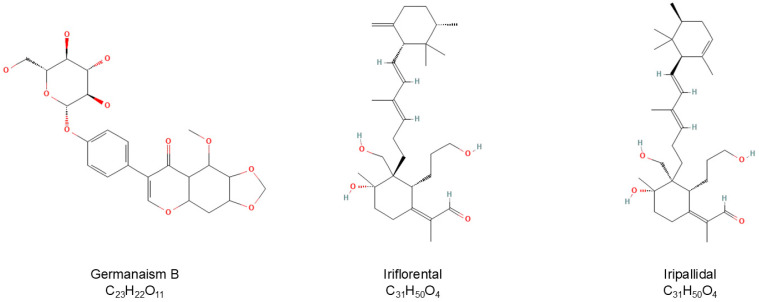
Chemical structures of germanaism B, iriflorental, and iripallidal.

Bioassay directed fractionation (BDF) is a common technique for fractionation of crude plant extracts and determination of bioactivity for collected fractions [49]. Active fractions can then be more fully characterized by instrumental analysis. To identify phytochemicals in the rhizome of *I. florentina* that influence melanogenesis, 90:10 ethanol/water extract was first separated using a reverse-phase HPLC system fitted with orthogonal detectors and the chromatographic effluent then directed to a fraction collector having a 96-well plate. Fractions were subsequently dried and tested for their effects on melanogenesis using mouse B16/F10 melanoma (B16) cells, with melanogenesis stimulated by 50 nM of α-MSH. B16 cells are a sensitive and reliable platform for screening small molecules that modulate melanogenesis. Figure 2 presents the alignment of LC-MS chromatographic data (Figure 2A) with bioassay results from % pigmentation (Figure 2B) normalized by cell viability. This comparison reflects the relative melanin content and melanogenic activity across the collected fractions. A prominent chromatographic peak at a retention time of 13.56 min, corresponding to fraction 41–43, showed increased melanogenesis activity and was identified as germanaism B (3). In contrast, fractions 90 and 93, eluting at 29.67 and 30.49 min were identified as Iriflorental (9) and Iripallidal (10), respectively, and showed inhibitory effects on melanin production. The chemical structures of germanaism B, iriflorental, and iripallidal are shown in Figure 3.

### 2.3. Confirmation of Melanogenesis-Modulating Activity of Purified Phytochemicals

**Figure 4:**
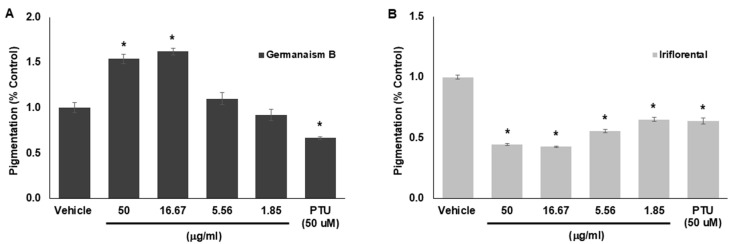
Effects of germanaism B (**A**) and iriflorental (**B**) on cellular melanin production. B16 cells were stimulated with 50 nM of α-MSH for 72 h in the absence or presence of the compounds. Relative Pigmentation (% Control) was calculated based on changes in optical density, normalized by cell viability. Data are presented as mean ± SD (*n* = 6). * Significant with respect to the Vehicle. Statistical analysis was performed using one-way ANOVA followed by Tukey’s post hoc test. Statistically significant values were defined as *p* < 0.05.

To validate the melanogenesis-stimulating and -inhibiting effects of the compounds identified above, purified germanaism B and iriflorental, were tested in B16 melanoma cells. Phenylthiourea (PTU) was included as a reference inhibitor to validate the assay. Figure 4 presents the percentage of pigmentation following treatment with various concentrations of the compounds. Germanaism B significantly stimulated melanogenesis at 50 and 16.67 μg/mL, increasing pigmentation by 53.6% and 62.3%, respectively. Lower treatment concentrations at 5.56 and 1.85 μg/mL, demonstrated reduced effects, indicating a peak stimulatory response at the higher doses rather than a linear does-dependent increase (Figure 4A). In contrast, iriflorental showed strong inhibitory activity on melanin production at all tested concentrations. At 50 and 16.67 μg/mL, it reduced pigmentation to 44.4% and 42.7%, respectively, compared to the vehicle control (set at 100%). Notably, its inhibitory effect at these concentrations even surpassed that of the reference compound, PTU at 50 μM. Unfortunately, iripallidal could not be evaluated due to challenges in purification. No cytotoxicity was observed for either compound at any of the tested concentrations. These findings confirm that the rhizome of *I. florentina* contains phytochemicals with opposing effects on melanogenesis, distinguished by their differing hydrophilic and lipophilic properties. This clear contrast in both bioactivity and solvent solubilities highlights the potential to selectively fractionate the extract into components with either stimulatory or inhibitory effects on melanogenesis, offering opportunities for targeted applications in opposite directions.

### 2.4. Evaluation of Hydrophilic and Lipophilic Extracts on Melanin Synthesis in 3D Skin Equivalent Models

To evaluate the effect of extract solubility on melanogenesis, 70:30 ethanol/water and chloroform extracts were tested using 3D skin equivalent tissues, MelanoDerm™ (MEL). MEL is a three-dimensional human skin tissue model that serves as a physiologically relevant alternative to animal testing, which is prohibited in many countries. This tissue consists of normal primary epidermal keratinocytes and melanocytes derived from a highly pigmented donor, seeded at a 10:1 ratio to closely mimic native human skin architecture. It is designed to promote melanin synthesis during the maintenance period and is used to evaluate the effects of test articles on melanogenesis.

**Figure 5:**
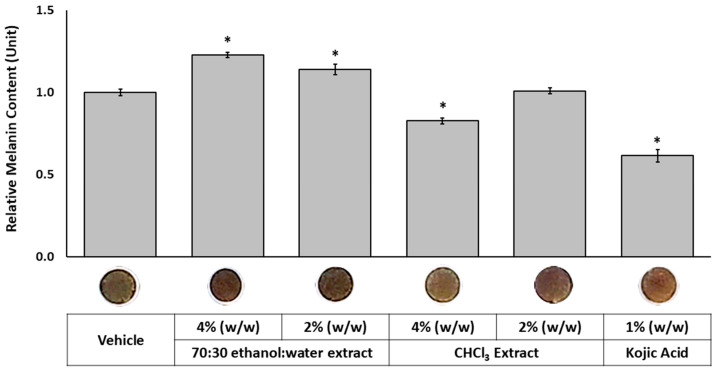
Effects of 70:30 ethanol/water and chloroform extracts on melanin content in a 3D skin equivalent model. MEL tissues were topically treated with 4%, 2% (*w*/*w*) of each extract, positive and vehicle controls, on alternate days for three weeks. At the end of the treatment period, tissue melanin content and viability were assessed. Representative macroscopic top-view images of the tissues were captured on the final day of treatment. Data are presented as mean ± SEM (*n* = 3) relative to vehicle control. * Significant with respect to the Vehicle. Statistics were analyzed with Microsoft Excel software utilizing a Student’s one-tailed *t*-test assuming equal levels of variance. Statistically significant values were defined as *p* < 0.05.

The extracts were topically applied to the skin tissues on alternate days over a three-week period at concentrations of 4% and 2% (*w*/*w*), respectively. On the last day of treatment, melanin contents were quantified, and top-view images of the tissues were taken to visually assess pigmentation (Figure 5). 70:30 ethanol/water extract increased melanin content by 22.6% and 13.9% when treated at 4% (*w*/*w*) and 2% (*w*/*w*), respectively. In contrast, 4% (*w*/*w*) treatment of chloroform extract decreased melanin content to 82.6% compared to vehicle control (100%). Macroscopic topical images of the tissues revealed visible differences in pigmentation after treatment. The 70:30 ethanol/water extract darkened the tissues, whereas the chloroform extract lightened them compared to the vehicle control, which is consistent with their respective effects on melanin content. As demonstrated by UV-HPLC (Figure 1) and BDF assay results (Figure 3), the two extracts are chemically distinct: the 70:30 ethanol/water extract is enriched in germanaism B, a compound known to stimulate melanin production, while the chloroform extract contains higher levels of iriflorental and iripallidal, both recognized inhibitors of melanogenesis. These results clearly indicate that different extraction processes, based on solvent polarity and solubility, can effectively separate melanogenesis-stimulating and -inhibiting compounds from the plant, enabling targeted approaches for different pigmentary conditions.

**Figure 6:**
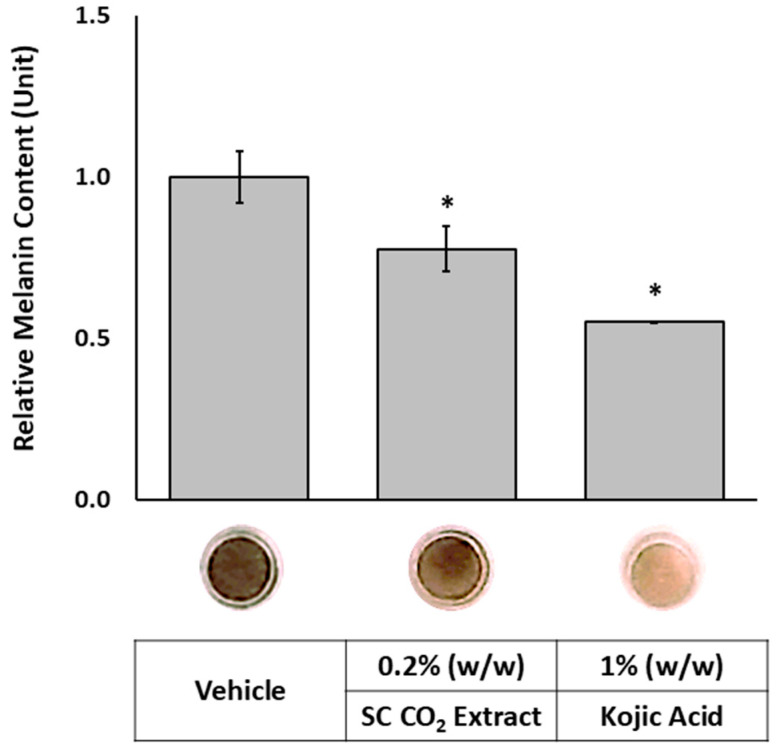
Effects of SC-CO_2_ extract on melanin content in a 3D skin equivalent model. MEL tissues were topically treated with 0.2% (*w*/*w*) of extract, positive and vehicle controls, on alternate days for three weeks. Data are presented as mean ± SEM (*n* = 3) relative to vehicle control. * Significant with respect to the Vehicle. Statistics were analyzed with Microsoft Excel software utilizing a Student’s one-tailed *t*-test assuming equal levels of variance. Statistically significant values were defined as *p* < 0.05.

**Figure 7:**
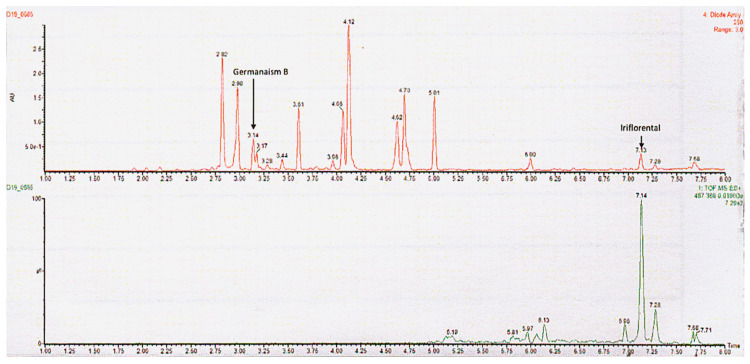
LC-UV at 260 nm (**top**) and LC-MS selected ion monitoring (**bottom**) chromatograms of SC-CO_2_ extract. Selected ion monitoring is at *m*/*z* 487.368, representing the protonated molecular ion for both iriflorental and iripallidal. The extract shows substantially reduced levels of germanaism B and enrichment of iriflorental and iripallidal.

As chloroform is classified as a carcinogen and prohibited in topical applications due to its toxicity, SC-CO_2_ extraction was adopted as a safer and environmentally friendly alternative. This method efficiently isolates lipophilic bioactive compounds from the plant without leaving harmful solvent residues. As shown in Figure 6, treatment with the SC-CO_2_ extract at 0.2% (*w*/*w*) reduced melanin content to 77.8% relative to the vehicle control (100%). A top-view image of the tissues confirmed a visible reduction in pigmentation following treatment. These findings suggest that the SC-CO_2_ extract achieved superior skin-lightening efficacy compared to the chloroform extract, despite being applied at a 20-fold lower concentration (0.2% vs. 4%, *w*/*w*). This enhanced performance is likely attributed to a greater yield of active lipophilic compounds. LC-UV and LC-MS chromatograms (Figure 7) show that the SC-CO_2_ extract contained substantially reduced levels of germanaism B and was enriched in iriflorental and iripallidal. Kojic acid at 1% (*w*/*w*), used as a positive control, also significantly reduced melanin content, supporting the validity of the study [7]. No cytotoxicity was observed with either extract treatment. Although bioactivity of germanaism B, iriflorental, and iripallidal is demonstrated, the mechanism of action underlying their effects on melanogenesis, including potential modulation of tyrosinase activity, MITF expression, or cAMP/α-MSH/MC1R signaling, were not explored and remain to be clarified. Overall, these results demonstrate that distinct extraction processes of *I. florentina* rhizome, optimized for hydrophilic or lipophilic compound isolation, can yield bioactives with differential effects on skin pigmentation, while offering safer and more sustainable options for topical applications.

## 3. Materials and Methods

### 3.1. Plant Material and Authetication

**Figure 8:**
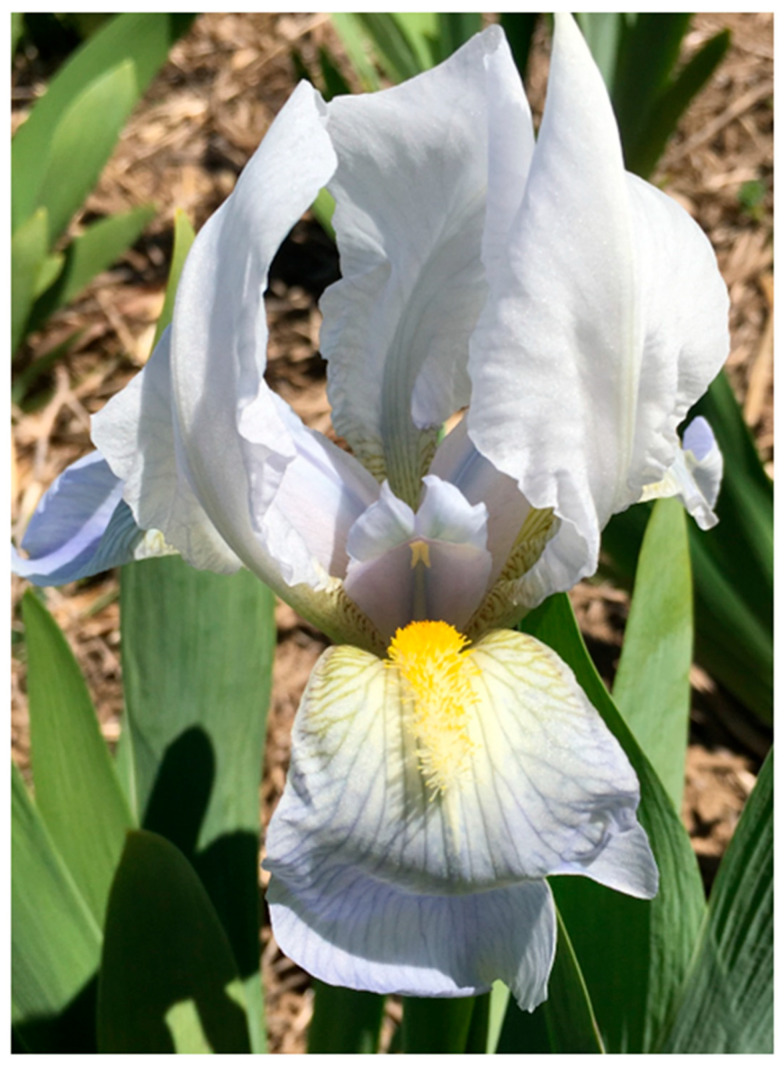
Photograph of an *I. florentina* L. specimen grown at Trout Lake Farm, WA, USA. Stigmas are pearl-colored, oval, and recurved, not revolute as in *I. germanica* and pollen grains are three-sided and elongated, proportionally longer than those of *I. germanica*. Photo credit: Franklin T. Johnson.

*Iris ×germanica* L. var. *florentina* Dykes (often referred to as *Iris florentina*) is a variety within the *Iris ×germanica* L. species. *I. florentina* rhizomes were sourced from a commercial nursery (Companion Plants, Athens, OH, USA) then grown and harvested in Amway-owned Trout Lake Farm-East located in Ephrata, WA, USA using organic farming practices (Figure 8). Verification of plant morphology characteristics was made using benchmarks provided by the American Iris Society to help authenticate as *I. florentina* when flower presented in the field. However, as current chemical test (such as HPTLC) and genetic (DNA) test did not conclusively differentiate *I. florentina* from other *I. germanica* varieties. A further comparative investigation using whole genome sequencing was conducted by collaborating with NSF International (Petaluma, CA, USA). Chloroplast DNA sequences were used to locate single nucleotide polymorphisms (SNPs) for several different non-*florentina I. germanica* cultivars and *I. florentina* specimens. Twenty-one positions were initially identified in an algorithmic search, and eleven were confirmed by observation. Primers were developed for five of these sequences and used for DNA testing. Of these, three SNPs successfully differentiated *I. florentina* specimens from other non-*florentina I. germanica* varieties tested.

### 3.2. Extraction

Rhizomes of *I. florentina* were harvested from the field and washed with water. After washing, hairy roots were manually removed using hand clippers. The rhizomes were then sliced into 2–5 mm thick sections using a kitchen knife until the majority of the rhizome tissue was separated from the plant and air-dried and milled to a fine powder.

Chloroform and hydroethanolic extracts (70:30 and 90:10 ethanol/water, *v*/*v*) were prepared following the same extraction procedure. Hydroethanolic solvents were prepared by mixing ethanol with distilled water in the appropriate ratios. Dried plant material (50 g) was added to 300 mL of each solvent (chloroform or hydroethanol) in separate flasks and stirred overnight at 22 ± 2 °C. The mixtures were then filtered through Whatman No. 1 filter paper, and the filtrates were concentrated under reduced pressure using a rotary evaporator. The collected extract was dried, stored at −80 °C, and later dissolved in DMSO at a concentration of 10 mg/mL for future use.

SC-CO_2_ extraction was performed using a Waters MV-10 ASFE^TM^ System (Waters Corporation, Milford, MA, USA). A 25 mL vessel was loaded with 13 g of dried and ground plant material. The extraction was conducted at a constant temperature of 55 °C and a pressure of 100 bar for 15 min. A flow rate of 8 mL/min CO_2_ and 2 mL/min ethanol cosolvent was used. Following the extraction, the system was depressurized gradually to atmospheric pressure. The collected extract was then dried, stored at −80 °C, and subsequently dissolved in DMSO at a concentration of 10 mg/mL for future use.

### 3.3. Reagents

Water, acetonitrile, and isopropanol were Optima LC-MS grade from Fisher Scientific (Pittsburgh, PA, USA). Formic Acid was LC-MS grade from Sigma-Aldrich (St. Louis, MO, USA). Mobile phase solvents were prepared as 0.1% formic acid solutions in water (Solvent A) or acetonitrile (Solvent B). DMSO was HPLC grade from Fisher Scientific (Pittsburgh, PA, USA). Germanaism B and iriflorental reference standards were isolated from methanol extracts of *I. florentina* rhizome by the Center for Natural Products Research, University of Mississippi, with structural confirmation by 1^H^ and 13C NMR, and high-resolution mass spectrometry.

### 3.4. Instrumentation

LC-MS analyses and BDF were performed using a Waters Synapt G2 instrument equipped with an Acquity H-class UPLC. Effluent from column was directed to a Photodiode Array Detector (PDA), and subsequently to the electrospray (ES) ionization source of the mass spectrometer. UV data was acquired from 200 to 800 nm at 1.2 nm resolution at a sampling rate of 5 scans/s. Mass spectral data was collected in both positive and negative ion modes at 20,000 resolving power (FWHM) from *m*/*z* 50–1200 at 0.25 scans/s. Within each run, alternating low energy (4 V) and high energy (20 V) collision induced dissociation scans were collected, using argon as collision gas (MSe mode). Leucine-enkephalin was used as the lock mass for accurate mass analysis. For presentation, data were displayed as either UV-Vis total wavelength chromatograms (sum of all acquired wavelength intensities) or high resolution MS selected ion monitoring mass chromatograms.

### 3.5. LC-MS Analysis

Sample extracts were prepared at 10 mg/mL in DMSO and sonicated for 20 min to solubilize. Process samples were diluted 1:10 in isopropanol. All samples were filtered through a 0.2 µm Whatman Anotop 25 syringe filter (Cytiva, Marlborough, MA, USA) into autosampler vials for assay. The column was a Waters Acquity HSS T3, 1.8 µm, 2.1 × 100 mm and the column oven was maintained at 40 °C. The solvent program was ramped linearly as follows: 90%A/10%B at time 0, to 100%B at 6 min, hold at 100%B to 8 min, then to 90%A/10%B at 8.01 min, and hold at 90%A/10%B for 10 min. A flow rate of 0.4 mL/min and an injection volume of 2 µL were used. Tentative identification of compounds was based on both the UV-Vis spectrum and mass spectrum of each peak compared to authenticated reference standards or on-line spectral databases, MoNA, https://mona.fiehnlab.ucdavis.edu (accessed on 1 March 2019) and MassBank, https://massbank.eu/MassBank/ (accessed on 1 March 2019) [50]. Accurate mass analyses of ions were performed to within 5 ppm for elemental composition verification.

### 3.6. Bioassay Directed Fractionation

Sample extracts were prepared at 50 mg/mL in DMSO and sonicated for 20 min. Samples solutions were then filtered through 0.2 µm Whatman Anotop 25 syringe filter into autosampler vials for assay. The LC-MS system was modified to incorporate a 10:1 splitter that was positioned between the UV detector and MS ion source. The majority of LC effluent was directed to a Waters Fraction Collector II fitted with a 96-deep well plate into which fractions were collected by time at intervals of 20 s/well for the first 32 min of each run (96 wells/run). The LC column used for BDF was a XBridge Shield RP18, 5 µm, 4.6 × 250 mm maintained at ambient temperature (22 °C). The solvent program was ramped linearly as follows: 95%A/5%B at time 0, to 100%B at 30 min, hold at 100%B to 32 min, then to 95%A/5%B at 32.1 min and hold 95%A/5%B until 36 min. The flow rate was 0.8 mL/min and an injection volume of 10 µL/injection was used (0.5 mg solids/injection). Effluent from four consecutive LC runs was collected per plate (2 mg solids/plate). Plates were then dried under nitrogen at 40 °C to remove acetonitrile and residual water was removed by freeze drying. Plates were stored at −80 °C until ready to assay for pigmentation response.

### 3.7. Cell Culture

B16-F10 (B16) cells obtained from ATCC (Manassas, VA, USA) were cultured at 37 °C in a humidified incubator with 95% air and 5% CO_2_. Cells were maintained in Dulbecco’s Modified Eagle’s Medium (DMEM) containing 4.5 g/L glucose, L-glutamine, and sodium pyruvate, supplemented with 10% fetal bovine serum (FBS) and 1% penicillin-streptomycin (100×). Subculturing was performed in T75 flasks every three to four days, ensuring confluency did not exceed 70% to maintain consistent growth. All experiments were conducted using cells between passages four and six [51].

### 3.8. Melanin Contents of B16 Cells

B16 cells (6000 cells/well) were pre-cultured in 96-well plates in DMEM supplemented with 10% FBS and 1% P/S for 24 h. For treatments, cells were treated with the vehicle or the testing articles in 0.2 mL DMEM-phenol red free medium supplemented with 10% FBS, 1% P/S, 2 mM L-glutamine, and 50 nM α-MSH (Sigma-Aldrich; St. Louis, MO, USA) for 96 h. The absorbance at 405 nm was measured. Cell viability was assessed by the MTT assay, in which cells were incubated with MTT (0.5 mg/mL) for 2 h, the resulting formazan crystals dissolved in isopropanol for 1 h, and absorbance measured at 570 nm [52,53]. The percentage of pigmentation was determined by normalizing the optical density (O.D) to the percentage of cell viability.

### 3.9. Skin Equivalent Tissue Maintenance and Treatment

The MelanoDerm™ (MEL) skin equivalents (Mattek Corp.; Ashland, MA, USA) derived from African American skin (MEL-B) were placed in an incubator containing 5% CO_2_ at 37 °C. The tissues were maintained in the LLMM medium. The testing articles were dissolved in vehicle control solution (90% PBS + 5% EtOH + 5% Propylene glycol) at desired concentrations. After 3 h of incubation, 25 μL of vehicle control, positive control, and the testing articles were applied topically to the skin equivalents every other day for three weeks. The tissues were washed with PBS between the treatments and replenished with 5 mL of fresh media every other day. On the final day, tissues were collected for visual analysis, melanin extraction, and cytotoxicity assessment [7,54]. Tissue viability was evaluated using the MTT assay, in which tissues were incubated with 1 mg/mL MTT solution for 3 h at 37 °C, followed by formazan extraction in isopropanol and measurement of absorbance at 570 nm. Viability was calculated relative to vehicle control to assess the cytotoxic effects of the treatments [55].

### 3.10. Melanin Content of Skin Equivalent Tissue

Melanin content in treated MEL-B was determined as previously reported [56]. Briefly, the frozen tissues were immersed in 0.38 mL of 1% SDS, 50 μM EDTA, and 10 mM Tris, pH 6.8, and 20 μL of protease K was added at 5 mg/mL. Digestion proceeded overnight at 45 °C, and an additional 20 μL of protease K was added for an additional 4 h of incubation. Then, 40 μL of 500 mM sodium carbonate and 10 μL of 30% H_2_O_2_ were added to the homogenates. The samples were incubated at 80 °C for 30 min and cooled to room temperature. Chloroform/methanol (2:1) mixture was prepared and 100 μL was added to each sample. After centrifugation at 10,000× *g* for 30 min, top phases were collected, and the optical density was measured at 450 nm. Synthetic melanin (Sigma-Aldrich; St. Louis, MO, USA) was subjected to the same procedure as a control, and a standard curve was constructed.

## 4. Conclusions

This study aimed to investigate the effects of extracts from the rhizome of *Iris florentina* on its ability to modulate melanogenesis. The BDF results demonstrated that the more hydrophilic fraction of the plant extract, which contains germanaism B, stimulated melanogenesis, while the more lipophilic fraction, enriched with iriflorental and iripallidal, inhibited melanin synthesis. These findings support a practical strategy for developing extracts that selectively separate these compounds, enabling targeted applications to either promote or suppress skin pigmentation. The hydrophilic fraction, obtained using a 70:30 ethanol/water extraction, was enriched with germanaism B and significantly stimulated melanin production in a 3D skin equivalent model. In contrast, the lipophilic fraction, extracted using chloroform and SC-CO_2_ methods, was enriched with iriflorental and iripallidal resulted in a clear reduction in melanin content. Notably, the supercritical CO_2_ extract achieved comparable depigmenting effects at a 20-fold lower concentration than the crude chloroform extract, while avoiding the use of toxic solvents. These results suggest that *I. florentina* rhizome is a promising botanical source for developing both skin-darkening and skin-lightening agents, depending on the extraction strategy employed. Future studies should elucidate the mechanisms of action through which germanaism B promotes, and iriflorental and iripallidal inhibit, melanogenesis. A better understanding of the mechanisms of action of these compounds could enable the identification of agents from other sources that target different yet complementary pathways, allowing synergistic combinations with germanaism B, iriflorental, or iripallidal to enhance melanogenesis regulation. In parallel, the effects of other phytochemicals identified in this study (Figure 1 and Table 1) should be further explored, as several compounds have demonstrated preliminary bioactivity, yet their potential skin health benefits remain largely unknown. Finally, efforts should be directed toward developing alternative, scalable, and cost-effective extraction methods, as current supercritical CO_2_ systems can be resource intensive. Establishing a unified extraction protocol capable of yielding both active fractions would further streamline ingredient production and improve commercial feasibility.

## 5. Patents

A patent application related to this work has been filed under the Patent Cooperation Treaty: Application No. PCT/US2022/031882.

## Figures and Tables

**Table 1:** Identified hydrophilic and lipophilic compounds in *I. florentina* rhizome extract by UV-HPLC.

Peak No.	Compound	CAS No.	t_R_ (min)	Molecular Formula	Measured [M+H]+	Calculated [M+H]+	Mass Error (ppm)	Activities
1	Irisolone 4′-O-diglucoside	50938-05-1	3.36	C_29_H_32_O_16_	637.1769	637.1763	0.9	-
2	Iridin	491-74-7	3.49	C_24_H_26_O_13_	523.1449	523.1452	−0.6	Anti-inflammatory [43], α-amylase inhibitory [42]
3	Germanaism B	123648-56-6	3.65	C_23_H_22_O_11_	475.1237	475.1240	−0.6	Antioxidant, antimicrobial [41,44]
4	Germanaism A	471271-89-3	3.69	C_24_H_24_O_12_	505.1343	505.1346	−0.6	Cytotoxic [43]
5	Irilone 4′-O-glucoside	50868-47-8	3.95	C_22_H_20_O_11_	461.1073	461.1084	−2.4	-
6	Irisolidone 7-O-glucoside	126308-74-5	4.11	C_23_H_24_O_11_	477.1398	477.1397	0.2	Antiproliferative [45]
7	Irigenin	548-76-5	4.57	C_18_H_16_O_8_	361.0914	361.0923	−2.5	Anti-inflammatory, α-amylase inhibitory, antioxidant, CYP1A-inhibitory [44]
8	Irisolidone	2345-17-7	5.4	C_17_H_14_O_6_	315.0868	315.0869	−0.3	Antioxidant, anti-inflammatory, antidiabetic, CYP1A-inhibitory [44]
9	Iriflorental	86293-26-7	7.52	C_31_H_50_O_4_	487.3780	487.3787	−1.4	Piscicidal [46]
10	Iripallidal	86293-27-8	7.68	C_31_H_50_O_4_	487.3779	487.3787	−1.6	Antiproliferative [47]

## Data Availability

The data presented in this study are available on request from the corresponding author due to company confidentiality policies and internal data handling procedures, and may be shared with permission from the company.

## Abbreviations

The following abbreviations are used in this manuscript:

SC-CO_2_	Supercritical carbon dioxide
LC	Liquid chromatography
MS	Mass spectrometry
UV	Ultraviolet
DHA	Dihydroxyacetone
α-MSH	Alpha melanocyte-stimulating hormone
MC1R	Melanocortin 1 receptor
SIK	Salt-inducible kinase
MITF	Microphthalmia-associated transcription factor
TRPM1	Transient receptor potential cation channel subfamily M member 1
IBMX	3-isobutyl-1-methylxanthine
cAMP	Cyclic adenosine monophosphate
PAR-2	Protease-activated receptor 2
UV-Vis	Ultraviolet-Visible
BDF	Bioassay directed fractionation
HPLC	High-performance liquid chromatography
PTU	Phenylthiourea
MEL	MelanoDerm™
MEL-B	MelanoDerm™ derived from African American skin
HPTLC	High-performance thin-layer chromatography
SNPs	Single nucleotide polymorphisms
DMSO	Dimethyl sulfoxide
NMR	Nuclear magnetic resonance
DMEM	Dulbecco’s modified eagle’s medium
FBS	Fetal bovine serum
MTT	3-(4,5-dimethylthiazol-2-yl)-2,5-diphenyltetrazolium bromide
